# Nutrition in the prevention and treatment of skeletal muscle ageing and sarcopenia: a single nutrient, a whole food and a whole diet approach

**DOI:** 10.1017/S0029665124007432

**Published:** 2024-10-17

**Authors:** Antoneta Granic, Avan A Sayer, Rachel Cooper, Sian M Robinson

**Affiliations:** 1 AGE Research Group, Translational and Clinical Research Institute, Faculty of Medical Sciences, Newcastle University, Newcastle upon Tyne, UK; 2 NIHR Newcastle Biomedical Research Centre, Newcastle upon Tyne Hospitals NHS Foundation Trust, Cumbria, Northumberland, Tyne and Wear NHS Foundation Trust and Newcastle University, Newcastle upon Tyne, UK

**Keywords:** Nutrition, Skeletal muscle, Sarcopenia, Ageing

## Abstract

Loss of skeletal muscle strength and mass (sarcopenia) is common in older adults and associated with an increased risk of disability, frailty and premature death. Finding cost-effective prevention and treatment strategies for sarcopenia for the growing ageing population is therefore of great public health interest. Although nutrition is considered an important factor in the aetiology of sarcopenia, its potential for sarcopenia prevention and/or treatment is still being evaluated. Nutrition research for sarcopenia utilises three main approaches to understand muscle-nutrition relationships, evaluating: single nutrients, whole foods and whole diet effects – both alone or combined with exercise. Applying these approaches, we summarise recent evidence from qualitative and quantitative syntheses of findings from observational and intervention studies of healthy older adults, and those with sarcopenia. We consider protein supplements, whole foods (fruits and vegetables) and the Mediterranean diet as exemplars. There is some evidence of beneficial effects of protein supplementation ≥ 0·8 g/kg body weight/d on muscle mass when combined with exercise training in intervention studies of healthy and sarcopenic older adults. In contrast, evidence for effects on muscle function (strength and physical performance) is inconclusive. There is reasonably consistent epidemiological evidence suggesting benefits of higher fruits and vegetables consumption for better physical performance. Similarly, higher adherence to the Mediterranean diet is associated with beneficial effects on muscle function in observational studies. However, intervention studies are lacking. This review discusses how current evidence may inform the development of preventive and intervention strategies for optimal muscle ageing and nutritional public policy aimed at combatting sarcopenia.

Skeletal muscle comprises about 30–40% of total body mass of the average adult person^(1)^, and is a multifunctional tissue^(2)^, essential for force generation and movement, breathing, temperature control^(1)^ and metabolism^(3)^. Specifically, skeletal muscle regulates glucose homeostasis^(3)^ and whole-body protein metabolism^(4)^ – serving as the primary site for glucose uptake and storage, and as the principal, active reservoir of amino acids for protein synthesis in other tissues^(4)^. Muscle health, described in terms of muscle mass and function (strength and physical performance), is affected by ageing processes^(5–8)^. After reaching a peak in the fourth decade of life^(8)^, muscle mass and strength start to decline gradually^(9,10)^, with the rate estimated to accelerate to 1–3% and 2·5–4% per year, respectively in later life^(8)^.

Although not fully explained, changes in skeletal muscle with age are linked to multiple molecular mechanisms, such as inflammation, dysfunction in mitochondria, oxidative stress and deregulation of nutrient sensing^(7,11–13)^. Declines in muscle strength and mass attributed to ageing characterise (primary) sarcopenia^(14,15)^, a progressive and generalised muscle disorder with a complex aetiology^(7,13,16,17)^ that has increasing prevalence with advanced age^(18)^. Depending on the definition and cut-offs applied for muscle mass and function, the global prevalence of sarcopenia varied from 10% to 37% in older adults aged ≥ 60 years, in a systematic evaluation of over 260 studies^(18)^. These estimates may change with the adoption of an inclusive definition of sarcopenia worldwide led by the Global Leadership Initiative in Sarcopenia (GLIS)^(19)^. The presence of sarcopenia and its components (low muscle mass, muscle strength and poor physical performance) associate strongly with adverse health outcomes in older adults, such as disability, frailty, hospitalisation and premature death^(14,20,21)^, accounting for a substantial health expenditure in the community and hospital settings^(22)^.

Although new drugs for sarcopenia are in development and existing drugs are being explored^(23)^, resistance exercise training (with/without nutritional supplementation) is considered the only effective non-pharmacological therapy for sarcopenia to date^(24,25)^. However, the anabolic response to exercise in older adults is less robust compared with younger adults^(26)^; the ability and willingness of older people to exercise may also be more limited^(24)^. This has focused interest on other influences on healthy ageing such as high-quality diet and nutrition to be explored for their effectiveness in the prevention and treatment of sarcopenia – especially those that are cost-effective and feasible for the growing older adult population across different communities and health care settings.

Ageing processes show marked inter- and intraindividual differences (heterogeneity) at the tissue and cell level within different body systems^(27)^, including muscle^(28)^, that are influenced by diverse internal and external factors^(29)^. For example, skeletal muscle demonstrates plasticity in response to internal (e.g. inflammation^(7,30)^) and external stimuli such as lifestyle, with exercise and nutrition playing important roles across the lifecourse^(24,25,29,31–33)^. The heterogeneity of muscle ageing and demonstrated muscle plasticity indicate the presence of modifiable factors that may influence the trajectory of muscle health with age and open the possibilities for the prevention and treatment of sarcopenia.

Since the conceptualisation of sarcopenia thirty years ago, research investigating nutrition-muscle relationships in observational and intervention studies in older adults has grown significantly, especially in the last decade^(34)^. Three main approaches have been utilised to investigate the role of nutrition in preserving muscle health and ameliorating its decline with age: single nutrients, whole foods and whole diets effects (Fig. 1(A)), alone or combined with exercise training^(25,31–35)^. However, despite the growth in research, there are still considerable gaps in understanding, especially for the links between nutrition and (incident) sarcopenia^(32,33)^, and no consensus about nutrition recommendations for the prevention and/or treatment of sarcopenia exists across the sarcopenia working groups worldwide^(36)^.

The primary scope of this review is to evaluate the most recent evidence from qualitative and quantitative syntheses of findings from observational and intervention studies that applied these three approaches in investigating skeletal muscle ageing/sarcopenia-nutrition relationships in older adults. To aid this novel evidence synthesis, we used three exemplars. Protein supplementation with/without exercise in healthy and sarcopenic older adults was used as an exemplar of single nutrients approach. Fruits and vegetables were used as an exemplar of whole foods approach, and the Mediterranean diet served as an example of a whole diet approach applied in observational studies.

## A single nutrient approach for the prevention and treatment of Sarcopenia

**Fig. 1:**
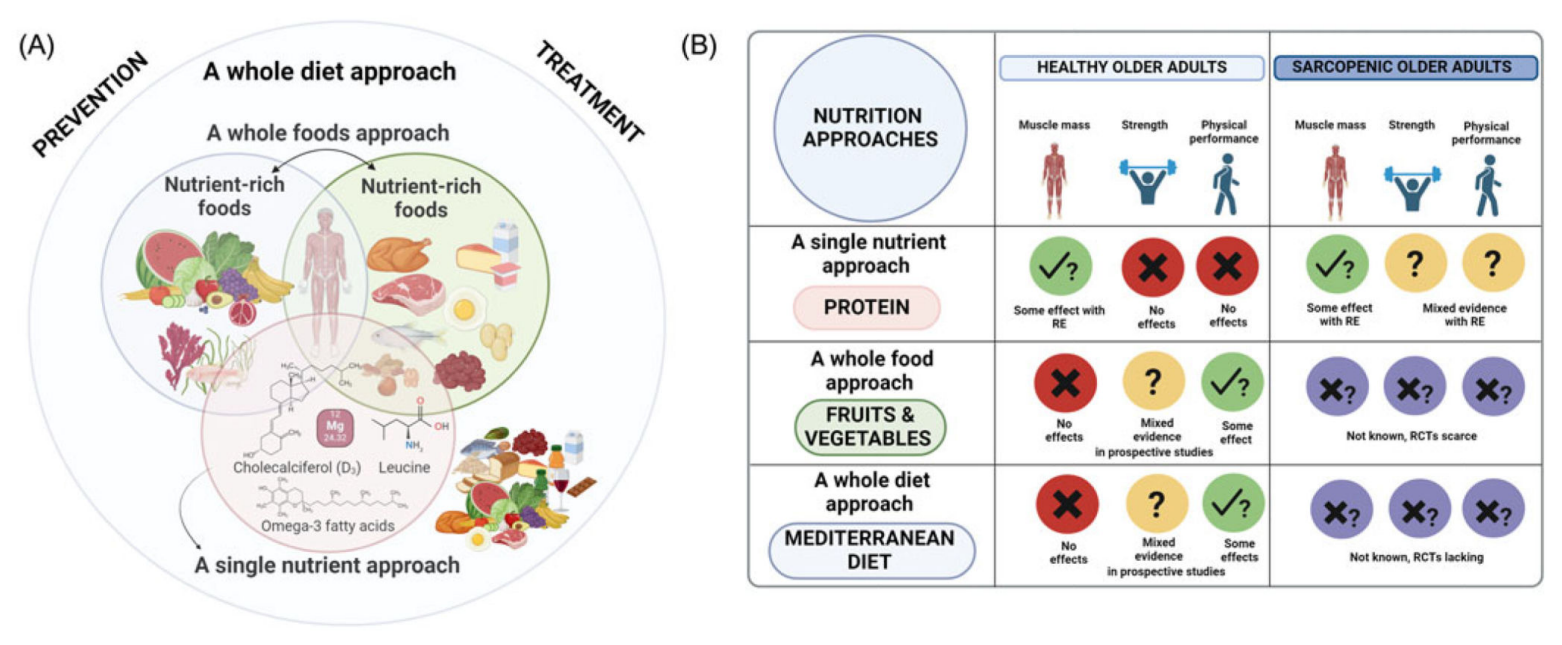
Summary of evidence from the three approaches applied in nutrition research for muscle health and sarcopenia. Created in BioRender. Granic, A. (2024) BioRender.com/x23y367 Recent evidence utilising a single nutrient, a whole food and a whole diet approach (panel A) was evaluated (panel B) from the latest qualitative and quantitative syntheses of observational and intervention studies in older adults with and without sarcopenia. Current evidence that may inform the development of preventive and intervention strategies for optimal muscle ageing and nutritional public policies aimed at combating sarcopenia is insufficient. Key: Red circles indicate evidence of no effect; yellow circles represent mixed, inconclusive evidence (i.e. evidence of effect/benefit or evidence of no effect); green circles indicate evidence of some effects/ benefits; purple circles indicate the absence of evidence or very scarce evidence of no effect for the selected outcomes. RE, resistance exercise; RCT, randomised controlled trial

Investigations aimed at preventing or reducing age-related losses of muscle mass and function that utilised a single nutrient approach have greatly concentrated on finding the optimal nutrient levels for muscle health, applied either singly or combined with exercise training^(25,31–57)^. These include protein (e.g. whey) and amino acids (e.g. leucine), *n*-3 fatty acids and vitamin D (Fig. 1(A)), with various combinations of protein supplementation with exercise being researched the most and systematically evaluated, including in meta- and network analyses^(25,31,40,42,44–56)^. In this section, we focus on protein. We briefly discuss the evidence for protein quantity, quality and timing for muscle health, summarising key findings from several recent quantitative syntheses of intervention studies (randomised controlled trials, RCTs) with protein, that were published in the last five years in older adults (aged ≥ 50 years) with and without sarcopenia.

### Dietary protein and supplements: quantity, quality and timing

#### Protein quantity

An adequate intake of dietary protein supplying essential amino acids is vital for muscle protein synthesis (MPS) and the maintenance of whole-body protein mass throughout the lifecourse^(33,37–39,58)^. Daily protein requirements for a healthy person aged ≥ 18 years, irrespective of sex and age, are estimated to range between 0·8–0·9 g/kg body weight (BW) based on nitrogen balance studies^(39)^ and are the basis of international nutritional guidelines. However, debate continues about the optimal protein intake for muscle health in older adults and whether it should be higher than 0·8 g/kg BW/d^(59)^, whilst considering other age-related changes influencing nutrition and skeletal muscle. These include changes in body composition^(60)^, digestive system (e.g. dysphagia and reduced gastric emptying)^(61)^, sensory system (taste and smell)^(62)^, energy requirements and expenditure^(63,64)^, appetite^(65)^ and the presence of ill health^(66,67)^. Additionally, it has been demonstrated that older adults are less sensitive to anabolic stimuli from protein for MPS in response to protein feeding compared with young adults, a process termed ‘anabolic resistance’^(68)^. For example, in a stable isotope tracer study older adults (aged 75 years) had a 16% lower post-prandial MPS rate after 20 g protein (casein) intake compared with young adults (aged 34 years), but comparable post-absorptive (basal) MPS rates^(69)^. However, in a study using the same methodology with healthy and sarcopenic older men (aged ≥ 70 years), similar basal and post-prandial MPS rates were observed after 21 g bolus intake of enriched (whey) protein^(70)^, indicating that the anabolic response in older sarcopenic muscle is not impaired.

Based on these and other evidence from cohort studies of ageing populations, several expert groups have increased dietary protein recommendations for older adults for the maintenance of muscle health, which may also prevent sarcopenia. For example, the recommendations of the PROT-AGE Study Group considered the health state and activity levels of an older person, proposing 1·0–1·2 g/kg BW/d of protein for healthy older adults, 1·2–1·5 g/kg BW/d for those with either chronic or acute illness, and 2·0 g/kg BW/d for malnourished older adults, and those with severe illnesses^(71)^. At least 1·2 g/kg BW of protein post-exercise has been recommended for active older adults to stimulate MPS^(71)^. However, in a meta-analysis conducted by the PROMISS (PRevention Of Malnutrition In Senior Subjects) consortium with four cohort studies and four national surveys from the EU and Canada in ~8100 older adults (aged ≥ 55 years), the prevalence of low protein intake was high, especially for the higher recommendations (e.g. 46·7% and 70·8% for 1·0 and 1·2 g/kg BW/d of protein, respectively)^(72)^. Similarly, according to the 2020–2025 Dietary Guidelines for Americans recommendations, about 30% of men and 50% of women aged ≥ 71 years do not consume enough protein-rich foods to meet the 0·8 g/kg BW/d requirement^(73)^.

#### Protein quality

Another aspect of dietary protein intake to be considered for recommendations to support muscle health is protein quality. Different measures have been used to characterise the biological value of protein needed to meet the metabolic demands of muscle tissue^(33)^. Lately, the Digestible Indispensable Amino Acid Score (DIAAS) methodology has been implemented for measuring digestibility of each amino acid in supplements, foods and diets, based on their ileal tract digestibility and accounting for the effect of food processing^(33,74)^. Current recommendations consider animal-based proteins as high-quality proteins with DIAAS of 100 because of their high content of essential amino acids, such as leucine, and higher digestibility compared with plant-based proteins (DIAAS range 80–85)^(33,75)^. However, production of plant-based proteins has lower environmental impact^(76)^ compared with animal-based proteins, and their anabolic properties for muscle could be enhanced by several strategies. These include having a higher protein intake, mixing different plant-based protein sources, or adding other anabolic stimuli (e.g. branched-chain amino acids such as leucine, *n*-3 fatty acids and exercise) to overcome their apparent inferiority to animal-based proteins for MPS^(77,78)^. Leucine and one of its metabolites, *β*-hydroxy-*β*-methyl butyrate are potent stimulators of MPS via, in part, the Akt-mTORC1-dependent (protein kinase B/mechanistic target of the rapamycin complex 1) signalling pathway and phosphorylation of 4E-BPs (the eukaryotic initiation factor 4E-binding proteins) and S6K1 (the ribosomal S6 protein kinase 1)^(79,80)^. It has been shown that the anabolic potential of protein-rich foods is largely influenced by their leucine content (i.e. when leucine is not delivered as a supplement), especially affecting the post-prandial MPS response in older adults^(81,82)^. However, the effects of protein supplements enriched with leucine or food fortification with leucine (with/without exercise) on muscle health (mass and function) were equivocal^(38,82)^. Overall higher leucine content of animal-based protein foods compared with plant-based foods^(38,82)^ should be considered when recommending *myoprotective* whole foods^(35,83)^ and the foods-first strategy for sarcopenia^(82,84)^.

#### Protein timing

The importance of protein timing and pattern of intake within a healthy diet (with/without exercise) has been researched lately, especially with the interest in chrono-nutrition and precision medicine approaches^(33,85,86)^. Skeletal muscle does not have an inactive reservoir for protein (amino acids) such as for glucose (via glycogen)^(3)^ but acts as an active protein store by incorporating dietary amino acids post-prandially^(4)^, which is used in response to stress (fasting, starvation, critical illness, traumatic injury)^(4)^. Thus, it has been suggested that per-meal protein intake and adequate anabolic response to each feed, as well as the distribution of protein across the meals, may be more important than total protein intake for muscle health in older adults^(87)^. Although various strategies have been tested to find the optimal protein distribution for ageing muscle (e.g. even, or skewed protein feeding across the meals), evidence to date is inconclusive^(87)^. A narrative review published in 2020 identified 12 observational and five intervention studies and concluded that even protein distribution may be an effective strategy to achieve a moderately high total protein intake in older adults who have low intakes (< 0·8 g/kg BW/d). For those with higher intake (0·8–1·3 g/kg BW/d), consumption of at least one meal with protein quantity sufficient to maximally stimulate MPS (30–40 g) is recommended for muscle health^(87)^. Breakfast could be considered as a high-protein meal based on the findings from a 2024 scoping review showing that higher protein consumption at breakfast may be beneficial for muscle mass but not for muscle strength in middle-aged and older adults^(88)^. Similarly, the PROMISS study findings from the longitudinal cohorts of older adults indicate benefits of consuming at least one meal with 30 g of protein/day to reach the minimum of 1·0 g/kg BW/d protein intake for muscle health^(89)^, regardless of the level of physical activity^(90)^.

In summary, protein as a single nutrient for muscle health in older adults has been well researched, however, findings are inconsistent and key recommendations are still lacking. Many narrative and systematic reviews, meta- and network analyses of observational and intervention studies have been published recently to understand the role of dietary protein and protein supplements (with/without exercise) in the prevention and treatment of sarcopenia in older adults^(e.g.(25,31–34,37–57,91,92))^. Here we summarise the main findings from most recent quantitative analyses of RCTs in healthy older adults and those with sarcopenia that may provide further insights about the protein-muscle health relationship and inform the strategies for sarcopenia treatment and prevention.

### Protein supplementation, muscle health and sarcopenia

#### Intervention studies with protein in healthy older adults

Optimal protein quantity to support muscle mass and function (muscle strength and physical performance), as key components of sarcopenia, in healthy adults (aged < 65 and ≥ 65 years) was investigated in a 2022 systematic review and meta-analysis of 74 RCTs (published by September 2020)^(31)^. Most studies tested the effect of additional protein ingestion (by cut-offs of < 1·2 g/kg BW/d, 1·2–1·59 g/kg BW/d, and ≥ 1·6 g/kg BW/d) with resistance exercise. Notably, daily protein ingestion and their sources varied across the studies, from 1–4·4 g/kg BW/d in the intervention and 0·8–2·3 g/kg BW/d in the placebo groups. Overall, the results indicate that increasing protein intake during resistance exercise may result in gains in lean body mass in 62 RCTs, an approximately 0·5–0·7 kg gain difference between the intervention and placebo groups. In older adults (≥ 65 years), the benefits were seen if consuming 1·2–1·59 g of protein/kg BW/d with exercise, and ≥ 1·6 g/kg BW/d with resistance exercise in those aged < 65 years. A small effect of ingesting additional protein was observed on lower-body strength in 47 studies of participants subjected to resistance exercise. However, the effect of ingesting additional protein (with/without resistance exercise) was unclear for grip strength and physical performance in younger and older adults^(31)^. The authors concluded that protein intake > 1·0 g/kg BW/d with resistance exercise may have beneficial effects on muscle mass, but not on other components of sarcopenia. This is consistent with the findings of the review by the Health Council of the Netherlands that examined the health effects of protein quantity above the population reference intake proposed by the European Food Safety Authority of 0·83 g/kg BW/ d^(93)^ for muscle health and sarcopenia components (lean body mass, muscle strength and physical performance^(41)^. This 2022 systematic review of 18 RCTs (published by April 2020) in older adults (aged ≥ 65 years), with an average habitual intake of ≥ 0·8 g/kg BW/d of protein from different sources and types (e.g. a mix of amino acids, protein supplementation), showed a possible beneficial effect of increased protein intake on lean body mass and muscle strength only when combined with exercise, and likely no effect on physical performance without exercise^(41)^. Taken together, the authors concluded that the evidence for increasing protein intake above 0·8 g/kg BW/d to improve muscle health in the general older adult population is low and not convincing.

A 2020 systematic review and meta-analysis of 65 RCTs (published by March 2019) focused on the timing of protein intake for muscle-related outcomes in healthy adults (< 55 and > 55 years)^(48)^. The source and quantity of protein supplementation varied across the studies with whey protein (alone or in combination with other nutrients) being mostly used, and the timing of protein intake tied to exercise (e.g. immediately after, before and after). Daily protein intake ranged from 0·9 g–2·3 g/kg BW. There was some evidence that protein supplementation was effective in improving muscle mass in younger and older adults which was not dependent on protein timing. Removing RCTs without exercise in older adults did not alter the findings. No beneficial effects of protein supplementation were found for muscle strength (grip strength and leg press)^(48)^. Overall, this evidence summary supports a possible beneficial effect of protein supplementation with exercise for muscle mass in healthy older adults, independently of the timing of protein intake.

A dose-response relationship between protein intake and increase in lean muscle mass was further evaluated in a 2020 systematic review and meta-analysis of 105 RCTs (published by May 2019) in healthy adults (aged 19–81 years)^(50)^. The multivariate-adjusted spline models revealed changes in lean muscle mass across a range of protein intake with or without exercise (e.g. a protein intake of 0·52–1·30 g/kg BW/d was associated with 0·06 kg (95% CI: 0·03, 0·08) mean increase in muscle mass without resistance exercise, and 0·40 kg (95% CI: 0·37, 0·43) with resistance exercise). An average effective dose of supplemental protein to increase muscle mass was determined as 0·17 g/kg BW/d. The results suggest that even a small increase in protein intake via supplementation may promote an increase in muscle mass with or without exercise^(50)^. In comparison, another systematic review and meta-analysis of 29 RCTs (published in November 2017) with nutrition without exercise in older adults (aged ≥ 65 years) assessed the dose, duration, frequency, timing and adherence to interventions^(53)^. The meta-analysis revealed an overall positive effect of amino acids, *β*-hydroxy-*β*-methyl butyrate, protein with amino acids supplementation on muscle mass, but not with protein alone, and protein with other nutrients. There was a great variability across the studies regarding the nutrition intervention characteristics and insufficient reporting about the treatment adherence, thus no conclusion could be drawn about the most effective intervention for muscle mass in older adults^(53)^. A recent 2024 systematic review of 14 RCTs (published in 2013–2023) examined nutrition strategies on sarcopenia components (mass, strength, physical performance) in middle-aged and older women^(56)^. Six out of nine RCTs reported that protein supplementation alone or in combination with other (non)nutrients (e.g. vitamin D, resveratrol) had a beneficial effect on muscle mass and mixed evidence for an effect on muscle function^(56)^. Taken together, protein supplementation interventions in middle-aged and older adults may have positive but limited effect on muscle mass, whereas benefits for muscle strength and physical performance are less clear.

The latest umbrella review of systematic reviews of nutrition interventions to improve muscle health in older adults (aged ≥ 65 years) was conducted by the Sarcopenia Guidelines Development group of the Belgian Society of Gerontology and Geriatrics^(42)^. Here, 15 reviews (published 2013–2017) were comprehensively assessed with the aim to generate recommendations based on the quality of evidence using the GRADE (Grading of Recommendations Assessment, Development, and Evaluation) method (i.e. certainty of evidence graded as *very low, low, moderate* and *high*). The supplements evaluated included protein supplementation, essential amino acids, leucine, *β*-hydroxy-*β*-methyl butyrate and others with or without exercise. Here we highlight the results for protein and leucine. Because of the low number and low quality of the reviews, the evidence supporting the recommendations was *low* to *moderate*. Specifically, there was *a low* level of evidence for a positive effect of protein supplementation for muscle mass, but no evidence for effects on muscle strength and physical performance. There was *a moderate* level of evidence for additive effect of protein supplementation in combination with resistance exercise for muscle mass in interventions lasting ≥ 24 weeks, and for muscle mass and strength in obese older adults, but no effects for physical performance. However, leucine supplementation alone has shown positive effects on muscle mass in older adults with sarcopenia (*moderate* level of evidence)^(42)^. The authors concluded that there was some evidence of beneficial effects of protein supplementation in combination with resistance exercise for muscle mass and strength in obese older adults, and for interventions lasting longer than 24 weeks, and leucine for sarcopenia.

In summary, despite extensive research on the effect of protein and amino acids supplementation (with/without exercise) on muscle in healthy older adults (≥ 65 years), there is still lack of information to inform dietary recommendations. Protein supplementation in combination with resistance exercise may be beneficial for muscle mass, especially in the interventions of longer duration. Protein intake > 0·8 g/kg BW/d in combination with exercise has a possibly positive effect on muscle mass, but not on other sarcopenia components. It is also noted that heterogeneity in study designs (e.g. for intervention: protein quality, quantity, timing), duration, outcome measures and populations studied, limited collation and quantitative synthesis of evidence.

#### Interventions with protein in older adults with sarcopenia

The following mixed conclusions have been obtained from the recent reviews and meta-analyses investigating the effectiveness of protein supplementation (with/without exercise) in older adults with sarcopenia^(40,43,46,52,54,91)^. Three meta-analyses evaluated RCTs investigating the effect of whey protein supplementation for improvements in sarcopenia components^(40,46,52)^. A 2023 meta-analysis of five RCTs (published by January 2023) in older adults with sarcopenia (average age 77·3 years) found a small increase in muscle mass and strength (grip strength) in interventions with whey supplementation and resistance exercise that did not reach the minimally important clinical difference. The qualitative synthesis of findings of whey supplementation for physical performance were mixed^(40)^. Another 2023 meta-analysis of 10 RCTs (published by December 2022) concluded that whey supplementation with exercise had no effect on sarcopenia components in sarcopenic older adults (aged ≥ 74 years)^(46)^. Positive results (effect sizes) for muscle strength were affected by the study durations and age of participants^(46)^. However, different results were obtained in a 2024 meta-analysis of 10 RCTs (published by June 2023) of whey supplementation (with/without resistance exercise) in older adults (aged ≥ 60 years) with sarcopenia living in the community or hospitalised^(52)^. Whey supplementation alone significantly increased muscle mass and physical performance (gait speed), and improved muscle strength (grip strength) when combined with resistance exercise in sarcopenic older adults, and promoted total protein and energy intake, and reduced inflammatory markers^(52)^. Similarly, another 2024 meta-analysis of 7 RCTs (published by January 2023) of protein supplementation with resistance exercise in community-dwelling older adults (aged ≥ 60 years) with sarcopenia showed significant improvements in muscle mass and strength (grip strength but not in chair rises)^(54)^. No improvements were seen in physical performance (gait speed)^(54)^.

To investigate further whether the combination of protein and resistance exercise is more effective in improving all components of sarcopenia compared with each intervention alone, a 2023 scoping review evaluated 59 studies (70% were RCTs; published in 2010–2023) in community-dwelling older adults (aged ≥ 60 years) with probable sarcopenia (low muscle mass or low muscle strength) or sarcopenia^(43)^. The results revealed that nutrition (protein, protein diet, protein supplements) with resistance exercise was the most frequent multicomponent intervention studied. This type of intervention and exercise-only intervention showed positive results most frequently for physical performance (e.g. gait speed), muscle mass and strength (grip strength). However, most studies lacked post-trial follow-up, missing out on valuable information about long-term benefits and potential hazards of these interventions^(43)^.

Lastly, several systematic reviews and meta-analyses combined RCTs with protein (with/without exercise) in healthy older adults and those with sarcopenia^(e.g. (44,49,51,55,92))^, and reported mixed results. For example, a 2023 systematic review and meta-analysis of 30 RCTs (published by June 2022) examined the effect of whey protein supplementation (with/without vitamin D, and in combination with resistance exercise or without) on sarcopenia components in older adults (aged ≥ 60 years)^(49)^. In subgroup analyses by sarcopenia status, whey protein supplementation significantly improved muscle mass and physical function in sarcopenic and frail older adults but had no effect on muscle health in healthy individuals. A 2024 network meta-analyses of 78 RCTs (published by July 2023) examined the effectiveness of protein supplementation with resistance exercise on sarcopenia components in community-dwelling, hospitalised and institutionalised older adults (aged ≥ 50 years) with acute and chronic conditions^(55)^. Of all protein sources identified, whey protein supplementation was the most efficient in enhancing the anabolic effect of resistance exercise on muscle mass, muscle strength (grip strength) and function (walking speed). These effects were moderated by sex, health conditions and supplement dose^(55)^. However, in contrast, no effects of nutrition interventions with whey protein or *β*-hydroxy-*β*-methyl butyrate (without exercise) were found in 12 RCTs (published by May 2021) on any of the sarcopenia components in subgroup analyses of healthy and sarcopenic older adults^(92)^.

Taken together, quantitative syntheses of numerous RCTs investigating the effects of protein as a single nutrient on sarcopenia components in older adults with sarcopenia showed benefits for muscle mass and strength when combined with exercise, and a possible benefit for physical performance. Whey protein with resistance exercise has consistently shown some positive effects across the trials. However, these effects may be dependent on the setting (community *v*. clinical), age and health of participants and protein dose.

In summary, there is some evidence for beneficial effects of protein supplementation with exercise for muscle health and sarcopenia in older adults. However, the conclusions of the systematic reviews, meta- and network analyses presented here need to be interpreted with caution. All studies have indicated a high variation in the methodological quality of the included RCTs, publication bias and differences across the RCT protocols (e.g. heterogeneity in protein supplementation (quantity, source, timing), outcome measures (e.g. non-standardised measurement of muscle mass) and the availability of data on baseline protein intake). Thus, a definite protocol of specific nutrition strategy with optimal dosage and duration of the protein intervention for the prevention and treatment of sarcopenia cannot be determined.

### A whole food approach for the prevention and treatment of Sarcopenia

#### Nutrient-rich foods with myoprotective potential: fruits and vegetables

Unlike the protein (amino acids)-muscle health relationship (a single nutrient approach) being examined in many qualitative and quantitative summaries of individual studies with older adults, the role of whole foods hypothesised to be beneficial for muscle health (*myoprotective*) has been rarely systematically evaluated^(32,35,83)^. Recently, we have summarised evidence from observational and intervention studies with nutrient-rich whole foods associated with the improvements in sarcopenia components in older adults^(35,38)^ and the risk reduction of incident sarcopenia in mid- and late adulthood^(32)^. The foods most frequently researched were protein-rich foods (e.g. dairy)^(94)^, and the foods consistently showing benefits for muscle health were antioxidant-rich foods (fruits and vegetables)^(35,83,95)^. In predominantly observational studies these foods were evaluated either singly^(94)^, or as multiple foods^(96)^, or their individual effects on sarcopenia components were assessed as a part of a heathy diet/dietary pattern^(97)^. Here, we have used fruits and vegetables as an exemplar of whole foods rich in nutrients (e.g. vitamins, minerals, fibre, proteins) and non-nutrients (e.g. biologically active phytonutrients such as lycopene and fisetin) implicated in general^(98–100)^ and muscle health^(101,102)^, through various cellular and molecular pathways, including antioxidative^(102,103)^ and anti-inflammatory pathways^(104)^.

The health benefits of higher consumption of variety of fruits and vegetables have been well established, with moderate to strong effects being reported for cardiovascular diseases, cancer and mortality risk reduction in numerous qualitative and quantitative synthesises^(105–108)^. As an essential part of a healthy, balanced diet across the lifecourse, fruits and vegetables are indorsed as a component of food-based dietary guidelines in over 100 countries to promote healthy eating habits and combat diet-related diseases in the population^(109)^. Compared with single nutrients, there is a greater understanding of a food-first approach among the general public to foster healthy ageing and longevity^(108)^. Biologically, whole foods such as fruits and vegetables may provide health benefits beyond the sum of those obtained from each constituent, in which nutrient and non-nutrient within the foods act synergistically and cumulatively on health outcomes. For example, higher habitual consumption of fruits and vegetables may increase the antioxidative capacity of the diet and counteract systemic low-grade inflammation (i.e. inflammaging) that arises through multiple molecular mechanisms, including oxidative stress and compromised antioxidative defence system that, in turn, activate pro-inflammatory signalling cascades^(110)^. Chronic inflammation is an established hallmark of ageing^(111,112)^ that, in connection with other hallmarks such as altered nutrient sensing^(113)^, drives many age-related processes and diseases^(114)^, including the inflammatory response in ageing muscle and sarcopenia^(7,30,115,116)^. Importantly, the Dietary Inflammation Index^®(117)^ have shown anti-inflammatory potential in reducing the risk of musculo-skeletal diseases^(118)^, including sarcopenia^(119,120)^. Thus, promoting higher habitual consumption of fruits and vegetables within a healthy diet for their antioxidative and anti-inflammatory properties may be one of the nutrition strategies to counteract detrimental effects of many age-related diseases with inflammaging and oxidative stress as underlying causes, such as sarcopenia.

#### Fruits and vegetables for the prevention and treatment of sarcopenia

In a 2020 systematic review, we evaluated evidence from 28 studies (19 observational and 9 interventional; published by March 2020) examining the relationship between individual whole foods (meat, fish, eggs, fruits and vegetables and non-liquid dairy with/without exercise), sarcopenia and sarcopenia components in adults aged ≥ 50 years^(83)^. The *myoprotective* potential of these foods was further examined in the most recent prospective studies and RCTs (without exercise) assessing later risk of sarcopenia and/or decline in sarcopenia components in mid- (< 60 years) and late adulthood in a 2024 narrative review (60–70 years)^(32)^. We also evaluated the latest scientific evidence (published between April 2022–November 2023) from studies of whole foods (protein-rich and antioxidant-rich foods; with/without exercise) in older adults aged ≥ 55 years^(35)^. The main conclusions from these qualitative syntheses were that largely consistent positive associations were observed between higher consumption of fruits and vegetables (total intake, fruits, or vegetable total intake) and measures of muscle health. These were based on observational studies (mostly cross-sectional), and evidence from RCTs was limited. Here we summarise key findings from these reviews and give examples of individual studies, concentrating on relevant prospective studies and RCTs that examined fruits and vegetables in relation to sarcopenia/sarcopenia components (with/without exercise) in older adults.

Our 2020 systematic review of individual *myoprotective* foods found positive associations between fruits and vegetables intake and physical performance, and moderate evidence for a role in preventing loss of muscle strength and sarcopenia, based solely on observational studies^(83)^. For example, an inverse dose-response relationship was found between baseline fruits and vegetables consumption and the risk of slow walking speed over 2·5 years in three independent cohorts of over 2900 community-dwelling older adults (aged ≥ 60 years) from Spain and France^(121)^. For participants consuming 1, 2, or ≥ 3 portions of fruits a day (1 portion = 120 g) compared with 0 portions, the odds of walking speed decline were 0·59 (95% CI: 0·27, 0·90), 0·58 (0·29, 0·86) and 0·48 (0·20, 0·75), respectively. Similarly, the corresponding odds for vegetables consumption (1 portion = 150 g) were 0·69 (0·42, 0·97), 0·56 (0·35, 0·77) and 0·52 (0·13, 0·92). However, no associations were found between fruits and vegetables consumption and muscle strength^(121)^. In a longitudinal study of over 1400 Australian post-menopausal women (aged ≥ 70 years), higher total vegetables (1 serving = 75 g) or fruits intake (1 serving = 150 g) was associated with reduced odds of muscle strength (grip strength) or physical performance decline (Timed Up-and-Go test) over 14·5 years of follow-up^(122)^. Compared with low vegetables intake (< 2 servings/d), a higher intake (≥ 3 servings/d) was associated with 31% lower odds of weak grip strength, whereas every 75 g increase in vegetables intake was associated with 12% lower odds of decline in Timed Up-and-Go test. For fruits, high (≥ 2 servings/d) compared with low (< 1 serving/d) intake was associated with 30% lower odds of grip strength, but not physical performance decline. Similar dose-response relationship between fruits and vegetables consumption and shared components of sarcopenia and frailty (low muscle strength and physical performance) was observed in a recent 2021 systematic review and meta-analysis of 14 observational studies (10 prospective)^(123)^. Mixed results were obtained in a study of over 430 middle-aged African American adults (aged ≥ 49 years) with low intake of fruits and vegetables at baseline^(124)^. At 6-year follow-up, higher intake of vegetables other than carrots, salads and potatoes was associated with stronger grip strength, whereas fruit juice was associated with grip strength decline^(124)^.

Further evidence for the role of fruits and vegetables in muscle health in mid-life was summarised in our 2023 review, specifically suggesting harmful effects of habitual low fruits and vegetables consumption in middle-aged individuals for the risk of muscle health decline in later life^(32)^. A longitudinal study of British civil servants, the Whitehall II study, assessed fruits and vegetables consumption on three occasions (1991–1993) when the participants were, on average, 50, 55 and 61 years old, before being assessed for muscle strength (GS) and physical performance (walking speed) in 2007–2009 at age of 66^(125)^. Low fruits and vegetables consumption (< 2 times/d) at every dietary assessment in mid-adulthood was associated with slower walking speed at follow-up. Furthermore, the accumulation-of-risk model with a cumulative fruits and vegetables score (i.e. the number of times low fruits and vegetables intake was reported over the three occasions) revealed the best model fit for longitudinal data, suggesting greater effects on muscle function with a longer duration of low fruits and vegetables consumption. These cumulative associations were independent of other unhealthy behaviours (smoking, low physical activity and nonmoderate alcohol drinking). In contrast, the cumulative associations with low fruits and vegetables and GS were weaker and attenuated by adjustment for other behavioural risk factors^(125)^.

Several studies examined the prospective associations between sarcopenia components and fruits and vegetables included in a dietary pattern (e.g. the Nordic Diet Score, Mediterranean diet) in older adults (aged ≥ 60 years), but found mixed results^(e.g. (97,126–128))^. For example, in the Helsinki Cohort Study of over 1000 older adults (born 1934–1944), habitual diet was assessed at mean age of 61 (2001–2004) to calculate the Nordic Diet Score score that included Nordic fruits (apples, pears and berries) and Nordic vegetables (tomatoes, cucumber, leafy vegetables, roots, cabbages and peas)^(126)^. Individual foods were examined for the prospective associations with physical performance (the Senior Fitness Test), muscle mass and strength (GS and leg strength) in men and women at age 71 years (2011–2013). Only consumption of Nordic fruits was positively associated with the Senior Fitness Test score in women, but not in men^(126)^. However, in another examination of the protective effects of Nordic fruits and vegetables on muscle mass and function in the same cohort, no associations were found at 10-year follow-up^(97)^.

A few studies examining the role of fruits and vegetables in muscle health in older adults have been published more recently and summarised in^(35)^. This latest qualitative synthesis for *myoprotective* roles of protein-rich (dairy) and antioxidant-rich (fruits and vegetables foods noted the dominance of observational studies (cross-sectional) and the lack of evidence from RCTs^(95)^. For example, in a 2020 systematic review and meta-analysis of 28 studies (19 observational (13 cross-sectional) and 9 RCTs; published between 2000–2020), the effect of antioxidant-rich foods (fruits and vegetables, beans, nuts, seeds, tea, cacao and oils) and antioxidant supplements on muscle health was investigated in adults aged ≥ 55 years^(95)^. Based on largely cross-sectional evidence, the authors found positive associations between higher fruits and vegetables consumption for muscle mass, strength, measures of muscle agility and mobility. Of five observational studies (four cross-sectional) examining the risk of sarcopenia in relation to antioxidant-rich foods (fruits and vegetables) or dietary patterns with higher consumption of these foods, only three found significant inverse associations. However, no RCTs examining the effect of fruits and vegetables in people with sarcopenia were reported^(95)^.

The lack of intervention studies with whole foods (fruits and vegetables), with/without exercise, for muscle health was also discussed in a 2022 review investigating lifestyle factors for the prevention and treatment of sarcopenia published between 2012–2022^(128)^. We have found one recent 6-month feasibility trial with 91 older adults (aged years ≥ 50 years) with habitually low fruits and vegetables consumption who were instructed to add 100 g/d (4 servings/d) of dried fruit to their diet to improve muscle mass and function^(129)^. No significant changes in physical performance, lean non-fat and non-bone lean muscle mass were observed after 6 months. Further examination of the RCTs registries for intervention studies with fruits and vegetables for muscle heath and sarcopenia revealed only a few new entries (e.g. Clinical Trial gov. NCT05863507, a trial of freeze-dried grape powder, a rich source of polyphenols, to mitigate sarcopenia in post-menopausal women), indicating that higher level of evidence is still absent. Taken together, there is a need for well-designed intervention studies, of sufficient duration, with whole foods (with/without exercise) to better validate their efficacy and mechanisms of action for the prevention and treatment of sarcopenia in a diverse population of older adults^(35,83,94,128)^.

In summary, there is reasonably consistent evidence mainly from observational studies showing benefits of higher consumption of fruits and vegetables for healthy muscle ageing. Higher daily intake of fruits and vegetables as a part of habitual diet may be beneficial for physical performance (in cross-sectional studies) and in preventing physical performance and muscle strength decline (in prospective studies) in middle-aged and older adults. Inconclusive evidence was found for their role in muscle mass. However, interventions with whole foods (fruits and vegetables) for muscle health are scarce, and those in older adults diagnosed with sarcopenia are lacking.

Potential health benefits of whole foods for ageing muscle should be considered in the context of other foods consumed within the diet/dietary pattern and the inevitable collinearity of individual foods (nutrients) in observational studies, which complicates the detection of an association (effect) of a single food and health outcomes. Because foods are consumed in combination, a low or high consumption of one (e.g. fruits and vegetables) could be an indicator of overall diet quality and the consumption of other foods that are either detrimental or beneficial for muscle heath. Therefore, a growing number of observational studies have used a whole diet approach to account for a complex interaction of foods and (non) nutrients and in diets and their influence on healthy ageing^(108)^.

### A whole diet approach for the prevention and treatment of Sarcopenia

#### Mediterranean diet

Of all healthy diets/dietary patterns investigated in nutritional epidemiology in relation to muscle ageing and sarcopenia in older adults, the Mediterranean diet has gained most scientific attention for potential health benefits. However, evidence is predominantly from observational studies^(32,130–142)^. Here we summarise evidence from several narrative and systematic reviews (with a few meta-analyses) of cross-sectional and prospective studies published recently (2017–2024), that investigated the Mediterranean diet in relation to sarcopenia in Mediterranean and non-Mediterranean populations^(130–139)^. These mostly qualitative syntheses assessed studies that used the Mediterranean diet-style indices only^(130–132,136–138)^ or evaluated the Mediterranean diet with other healthy dietary patterns^(32,133–135,139)^. Evidence from prospective studies was scarce^(135)^, especially studies with repeat dietary assessments^(32,143)^, whereas RCTs were lacking^(134,137)^. Only two included meta-analyses because of a high heterogeneity across the studies (e.g. differences in the Mediterranean diet indices (exposure), muscle-related outcomes, and their measurements, low number of studies with sarcopenia, and variations in adjustments for confounding). Overall, reasonably consistent positive results were found between a higher Mediterranean diet adherence and muscle function (walking speed, mobility) in cross-sectional studies and less decline over time in prospective studies. Mixed results were found for other sarcopenia components, muscle strength (grip strength) and muscle mass, and inconclusive evidence for sarcopenia in a few observational studies. Without RCTs, cause and effect conclusions for the Mediterranean diet in muscle health (prevention and treatment of sarcopenia) with ageing cannot be determined.

#### Characteristics of Mediterranean diet

The Mediterranean diet represents a healthy dietary pattern that has been extensively studied in relation to various health outcomes^(142)^, including healthy ageing and longevity^(141)^. The original Mediterranean diet represents a traditional diet of populations living in the Mediterranean Basin during the 50s and 60s in the last century^(142)^. Despite changes in the diet composition in the last decades, the Mediterranean diet, as a plant-based diet, is considered environmentally sustainable^(143)^. The traditional Mediterranean diet is characterised by a low consumption of red meats and meat products, moderate consumption of fish, eggs, poultry, fermented dairy (cheese and yoghurt) and high consumption of plant foods (fruits, vegetables, legumes, tree nuts and cereals) and extra-virgin olive oil as the main source of fat. Red wine is consumed moderately during meals.

To measure adherence to the Mediterranean diet-style diet in different populations, various indices/scores have been developed. For example, there were 22 indices by 2015 that differed in their scoring systems and composition (e.g. Mediterranean Diet Scale: 9 components, range 0–9; Mediterranean Lifestyle Index: 28 components, range 0–28)^(144)^. However, the main principles of the Mediterranean diet – its plant-based core and a low red meat consumption (positive components) – have been preserved across the indices^(144)^.

The Mediterranean diet is a rich source of numerous nutrients and non-nutrients, such as vitamins (carotenoids, C, D, E, folate), minerals (K, Mg, Fe, Se, Ca), mono- and polyunsaturated fatty acids (MUFA and PUFA) and phytochemicals that have antioxidative and anti-inflammatory properties^(134,145)^. We have hypothesised that these (non)nutrient may have a direct effect on the ageing muscle (i.e. *myoprotective* effect) by acting synergistically and cumulatively on the pathophysiological mechanisms of sarcopenia^(7,13)^, and an indirect effect by reducing the risk of age-related conditions related to sarcopenia^(134)^. Other benefits of the Mediterranean diet may involve lesser acidity and favourable acid-base balance of the diet, which have been implicated in muscle health in older adults^(146,147)^.

#### Mediterranean diet for the prevention and treatment of sarcopenia

##### Evidence summary from narrative and systematic reviews

Here we summarise key findings and conclusions of several recent narrative and systematic reviews that investigated the associations between sarcopenia components and sarcopenia in various populations of middle-aged and older adults (aged ≥ 45 years)^(130–139)^. Four reviews (published 2017–2018) have been described in detail in our 2019 systematic review of dietary patterns, muscle ageing and sarcopenia^(134)^. Briefly, in a 2017 systematic review of observational studies (published in 2016) examining the role of the Mediterranean diet in musculoskeletal health across the lifecourse, only two studies (one cross-sectional and one prospective) in older adults were included^(130)^. Positive cross-sectional associations were reported between the Mediterranean diet, muscle mass and leg power, but no associations were found in the prospective study^(130)^. A 2017 narrative review of the role of the Mediterranean diet in frailty and sarcopenia, synthesised evidence from 12 observational studies (7 assessed sarcopenia or components of sarcopenia/sarcopenic symptomology; published 2008–2017) in older adults (aged ≥ 55 years)^(131)^. All studies (cross-sectional and prospective) reported lower risks of sarcopenic symptomology with greater adherence to the Mediterranean diet (i.e. lower extremity functioning and mobility). A 2018 systematic review and meta-analysis of 12 studies (8 prospective; published 2011–2017) investigated the relationship between adherence to the Mediterranean diet, frailty, functional disability and sarcopenia in older adults (aged ≥ 60 years)^(132)^. Only two prospective studies of sarcopenia were included and showed no association between the Mediterranean diet and sarcopenia risk. However, cross-sectional studies indicated better sarcopenia components with higher Mediterranean diet scores. Also, higher adherence to the Mediterranean diet was associated with a lower risk of frailty and functional impairment (e.g. assessed by physical component sub-scale of SF-12)^(132)^. Another 2018 systematic review of the association between diet quality (pre-defined or data-driven dietary indices) and sarcopenia in middle-aged and older adults (aged ≥ 50 years), included 26 studies (published by 2016) and found strong observational evidence for the association between ‘heathier’ diets (including the Mediterranean diet) and lower risk of decline in physical performance (e.g. walking speed), but not for decline in muscle strength^(133)^. Evidence from cross-sectional studies for other sarcopenia components and sarcopenia was weak^(133)^.

In our 2019 systematic review, along with the summary of evidence from previous reviews^(130–133)^, we have included additional studies (published 2017–2019) that further corroborated the positive associations between the Mediterranean diet scores and better physical performance (walking speed and Timed Up-and-Go test) in cross-sectional but not in prospective studies^(134)^. The main conclusions of this review were that only a few studies have investigated the role of the Mediterranean diet in the aetiology of sarcopenia, but a number of studies have explored the relationship between components of sarcopenia and a decline in physical function. Specifically, higher adherence to the Mediterranean diet was positively associated with lower extremity functioning, mobility and better walking speed over time. No associations were found for the measures of upper-body muscle strength in most of the studies. The results suggest that, whilst the Mediterranean diet may not improve muscle strength in older adults, higher adherence to the Mediterranean diet-style diets may be beneficial for mobility and general physical functioning. Based on this summary, the Mediterranean diet may have the greatest *myoprotective* potential compared with other healthy diets. However, prospective studies with longer duration are needed to support future clinical trials of *myoprotective* dietary patterns in older adults. Differences in findings could be explained by heterogeneity across the studies, especially in the Mediterranean diet indices used in different populations. To date, no univocal definition of the Mediterranean diet and its dietary score exists, which should be based on modern understanding of the Mediterranean diet^(143)^ to advance the field of nutritional epidemiology and sarcopenia^(108)^.

Further evidence summary from a 2021 systematic review of diet quality and sarcopenia in middle-aged and older adults (age ≥ 45 years) was based on 14 prospective studies (7 with the Mediterranean diet; published by 2020)^(135)^. Mixed results were reported suggesting benefits of the Mediterranean diet for muscle mass, but inconclusive evidence for muscle strength (grip strength). The evidence for physical performance (walking speed, the risk of developing mobility disability) was also mixed, with some studies reporting positive and others no associations (especially in women). Only a few studies examined the associations between the Mediterranean diet and sarcopenia and found nil results^(135)^. The authors concluded that there is conflicting evidence for the association between the Mediterranean diet, sarcopenia components and sarcopenia^(135)^. Another 2021 systematic review and meta-analysis of 53 observational studies (19 cross-sectional, 35 prospective studies; published by May 2021), investigated the association between the Mediterranean diet adherence and cognitive and physical functioning in older adults (aged ≥ 60 years)^(136)^. Eight cross-sectional and 10 prospective studies examined the link between the Mediterranean diet and components of sarcopenia (walking speed, Timed Up-and-Go test, grip strenght, Short Physical Performance Battery). In a meta-analysis of cross-sectional studies, a high adherence to the Mediterranean diet was associated with better walking speed (Standard Mean Difference 0·42 (95% CI: 0·12, 0·72)) and knee muscle strength (0·26 (95% CI: 0·17, 0·36)). No associations were found for GS and mobility. Equally, no prospective associations were found in meta-analysis of five studies linking the Mediterranean diet adherence to incidence of mobility problems^(136)^. The latest 2023 systematic review of the role of the Mediterranean diet in the prevention of sarcopenia (components), included 10 studies (6 prospective; published 2000–2022) in healthy older adults (age ≥ 65 years)^(137)^. Both positive and negative associations were reported for muscle strength (grip strength) in four studies. Of six studies (five prospective) investigating the association between the Mediterranean diet and muscle function (walking speed, Short Physical Performance Battery, squat test), all but one reported positive association. No associations were observed between the Mediterranean diet and sarcopenia in a few included studies^(137)^.

None of the summarised reviews reported RCTs with the Mediterranean diet in healthy or sarcopenic older adults.

##### Additional evidence from individual studies

We have identified three recent individual studies that investigated the associations between the Mediterranean diet and sarcopenia^(148–150)^ classified with the European Working Group on Sarcopenia in Older People (EWGSOP2) definition^(14)^. A cross-sectional study of 2963 participants (mean age 72·8 ± 5·7 years) enrolled in the Longevity Check-up 7+ project, investigated the associations between the Mediterranean diet (a modified Medi-Lite categorised as low (≤ 8), good (9–11), or high (≥ 12)) and probable sarcopenia (low GS; < 27 kg in men, and < 16 kg in women)^(148)^. Those with lower Mediterranean diet score (low adherence) had higher prevalence of probable sarcopenia (25·9%) compared with those with good and high scores (19·1% and 15·5%, respectively). In the fully adjusted models, good (OR 0·71 (95% CI: 0·55, 0·92)) and high (0·60 (95% CI: 0·44, 0·81) adherence to the Mediterranean diet was associated with lower odds of probable sarcopenia^(148)^. Further analyses of a combined effect of aerobic training and the Mediterranean diet score in 491 participants (mean age 72·7 ± 5·7 years) with sarcopenia (low grip strength and appendicular muscle mass), revealed no associations with sarcopenia or sarcopenia components^(149)^. In a cross-sectional study of 528 Italian adults (aged ≥ 50 years) attending health screening checks at a local hospital, diets in the lowest third of the Mediterranean diet pattern (detected by principal components analysis) were associated with increased odds of probable sarcopenia (2·38 (95% CI: 1·05, 5·37)) and sarcopenia (9·69 (95% CI: 1·41, 66·29)) compared with the highest third^(150)^. Because of wide confidence intervals (CI), the result for sarcopenia needs to be interpreted with caution.

Taken together, although the evidence for the benefits of a higher adherence to the Mediterranean diet for sarcopenia (sarcopenia components) in prospective studies of older adults are emerging^(135,136)^, positive associations summarised in recent reviews (2017–2024) come largely from cross-sectional studies, and their limitations must be considered when interpreting the results (e.g. reverse causality). Higher adherence to the Mediterranean diet was associated with lower extremity functioning, mobility and better walking speed. The associations with sarcopenia were mostly non-significant, although evidence is limited. The heterogeneity of evaluated studies was high, and only a few meta-analyses of cross-sectional studies with the Mediterranean diet were conducted. No clinical trials were reported.

### Summary and future direction

In this review, we have evaluated the most recent evidence from qualitative and quantitative syntheses of observational and intervention studies that used a single nutrient (protein supplementation), a whole food (fruits and vegetables), or a whole diet (the Mediterranean diet) approach to investigate nutrition-muscle ageing relationships in older adults with and without sarcopenia (Fig. 1(A)). Key findings are summarised in Fig. 1(B).

Overall, there is some evidence of associations between protein and amino acids (leucine) supplementation and muscle in healthy older adults (aged ≥ 65 years). Protein intake above 0·8 g/kg BW/d in combination with exercise may be beneficial for muscle mass, especially in the interventions of longer duration^(31,41,42,48,53,56)^, but not for other sarcopenia components^(41)^. In older adults with sarcopenia, protein (whey) supplementation in combination with exercise training showed benefits for muscle mass and muscle strength^(43,49,51,52,54)^, and physical performance in some meta-analyses^(43,52)^. However, there is considerable heterogeneity in study protocols and in the quality of trials and limited quantitative syntheses of evidence and, key recommendations for protein are still lacking. Debate continues about the quantity, quality, frequency and timing of protein consumption for the prevention and treatment of sarcopenia^(31,38,39)^. Future high-quality intervention studies are needed to inform the development of nutrition strategies that specify optimal levels of protein intake for the prevention and treatment of sarcopenia in older adults.

There is reasonably consistent evidence from predominantly observational studies of associations between higher consumption of fruits and vegetables and better physical performance in cross-sectional studies^(35,83)^ and the prevention of physical performance and muscle strength decline in prospective studies^(32,125)^. These observational results need to be tested in interventions with whole foods (fruits and vegetables) in older adults with and without sarcopenia as there are still considerable gaps in nutrition research about the role of whole foods in heathy muscle ageing. This is especially as focusing on whole foods as a strategy to promote overall^(105,106)^ and muscle health may be more acceptable to older adults and health professionals compared with single nutrients^(151,152)^.

For a whole diet approach with the Mediterranean diet as an exemplar of healthy and sustainable diet^(140–143)^, reasonably consistent positive results were found between the adherence to the Mediterranean diet and better muscle function (e.g. lower-body function) in cross-sectional studies^(131,132)^, and slower decline in function in a few prospective studies^(133,134)^. Mixed results were found for other sarcopenia components, muscle strength and muscle mass and there was inconclusive evidence for sarcopenia in a few observational studies^(135–137)^. Importantly, there were few prospective studies with repeated dietary assessments across adulthood to inform preventive strategies^(32,143)^, and no clinical trials^(134,137)^. Therefore, there are still big gaps in knowledge and a need for long-term RCTs to unravel the associations between the Mediterranean diet and muscle health with ageing. Additionally, scientific consensus on defining compliance with the Mediterranean diet (indices) based on the latest understanding of the Mediterranean diet^(143)^ is needed, with external validation in different populations^(144)^ in well-conducted observational studies to reduce heterogeneity across studies.

In conclusion, since the conceptualisation of sarcopenia approximately thirty years ago, the three approaches to the characterisation of diet (from single nutrients to whole diets) described here have amassed a large body of literature aimed at the development of preventive and intervention strategies for healthy muscle ageing. However, we have found considerable gaps in knowledge as required to achieve full consensus about nutrition recommendations for sarcopenia that can inform public policy^(36,57)^. There was some evidence for the benefits of protein supplementation ≥ 0·8 g/kg BW/d with exercise for muscle mass in intervention studies with healthy older adults and those with sarcopenia. However, this evidence is still insufficient for recommending a nutrition strategy with optimal protein intakes for the prevention and/or treatment of sarcopenia. There was reasonably consistent evidence for benefits of higher consumption of fruits and vegetables and higher adherence to the Mediterranean diet for physical functioning in predominantly observational studies, but a substantial lack of interventions, particularly in adults with sarcopenia (Fig. 1(B)). Current efforts to harmonise the operational definition of sarcopenia by GLIS^(19)^ will greatly contribute to advancing our understanding of nutrition-sarcopenia relationships to inform public health recommendations for optimal skeletal muscle ageing in the future.

## References

[1] Frontera WR, Ochala J (2015). Skeletal muscle: a brief review of structure and function. Calcif Tissue Int.

[2] Mukund K, Subramaniam S (2020). Skeletal muscle: a review of molecular structure and function, in health and disease. Wiley Interdiscip Rev Syst Biol Med.

[3] Merz KE, Thurmond DC (2020). Role of skeletal muscle in insulin resistance and glucose uptake. Compr Physiol.

[4] Wolfe RR (2006). The underappreciated role of muscle in health and disease. Am J Clin Nutr.

[5] Tieland M, Trouwborst I, Clark BC (2018). Skeletal muscle performance and ageing. J Cachexia Sarcopenia Muscle.

[6] Wilkinson DJ, Piasecki M, Atherton PJ (2018). The age-related loss of skeletal muscle mass and function: measurement and physiology of muscle fibre atrophy and muscle fibre loss in humans. Ageing Res Rev.

[7] Granic A, Suetterlin K, Shavlakadze T (2023). Hallmarks of ageing in human skeletal muscle and implications for understanding the pathophysiology of sarcopenia in women and men. Clin Sci (Lond).

[8] Mitchell WK, Williams J, Atherton P (2012). Sarcopenia, dynapenia, and the impact of advancing age on human skeletal muscle size and strength; a quantitative review. Front Physiol.

[9] Dodds RM, Syddall HE, Cooper R (2016). Global variation in grip strength: a systematic review and meta-analysis of normative data. Age Ageing.

[10] Janssen I, Heymsfield SB, Wang Z (2000). Skeletal muscle mass and distribution in 468 men and women aged 18–88 years. J Appl Physiol (1985).

[11] Sartori R, Romanello V, Sandri M (2021). Mechanisms of muscle atrophy and hypertrophy: implications in health and disease. Nat Commun.

[12] Lai Y, Ramírez-Pardo I, Isern J (2024). Multimodal cell atlas of the ageing human skeletal muscle. Nature.

[13] Wiedmer P, Jung T, Castro JP (2021). Sarcopenia – molecular mechanisms and open questions. Ageing Res Rev.

[14] Cruz-Jentoft AJ, Bahat G, Bauer J (2019). Sarcopenia: revised European consensus on definition and diagnosis. Age Ageing.

[15] Cruz-Jentoft AJ, Sayer AA (2019). Sarcopenia. Lancet.

[16] Larsson L, Degens H, Li M (2019). Sarcopenia: aging-related loss of muscle mass and function. Physiol Rev.

[17] Zhong Q, Zheng K, Li W (2023). Post-translational regulation of muscle growth, muscle aging and sarcopenia. J Cachexia Sarcopenia Muscle.

[18] Petermann-Rocha F, Balntzi V, Gray SR (2022). Global prevalence of sarcopenia and severe sarcopenia: a systematic review and meta-analysis. J Cachexia Sarcopenia Muscle.

[19] Kirk B, Cawthon PM, Arai H (2024). The conceptual definition of sarcopenia: Delphi consensus from the Global Leadership Initiative in Sarcopenia (GLIS). Age Ageing.

[20] Yuan S, Larsson SC (2023). Epidemiology of sarcopenia: prevalence, risk factors, and consequences. Metabolism.

[21] Beaudart C, Zaaria M, Pasleau F (2017). Health outcomes of sarcopenia: a systematic review and meta-analysis. PLoS One.

[22] Norman K, Otten L (2029). Financial impact of sarcopenia or low muscle mass – a short review. Clin Nutr.

[23] Rolland Y, Dray C, Vellas B (2023). Current and investigational medications for the treatment of sarcopenia. Metabolism.

[24] Hurst C, Robinson SM, Witham MD (2022). Resistance exercise as a treatment for sarcopenia: prescription and delivery. Age Ageing.

[25] Shen Y, Shi Q, Nong K (2023). Exercise for sarcopenia in older people: a systematic review and network meta-analysis. J Cachexia Sarcopenia Muscle.

[26] Endo Y, Nourmahnad A, Sinha I (2020). Optimizing skeletal muscle anabolic response to resistance training in aging. Front Physiol.

[27] Tian YE, Cropley V, Maier AB (2023). Heterogeneous aging across multiple organ systems and prediction of chronic disease and mortality. Nat Med.

[28] Kirkeby S, Garbarsch C (2000). Aging affects different human muscles in various ways. An image analysis of the histomorphometric characteristics of fiber types in human masseter and vastus lateralis muscles from young adults and the very old. Histol Histopathol.

[29] Brook MS, Wilkinson DJ, Phillips BE (2016). Skeletal muscle homeostasis and plasticity in youth and ageing: impact of nutrition and exercise. Acta Physiol (Oxf).

[30] Liang Z, Zhang T, Liu H (2022). Inflammaging: the ground for sarcopenia?. Exp Gerontol.

[31] Nunes EA, Colenso-Semple L, McKellar SR (2022). Systematic review and meta-analysis of protein intake to support muscle mass and function in healthy adults. J Cachexia Sarcopenia Muscle.

[32] Robinson S, Granic A, Cruz-Jentoft AJ (2023). The role of nutrition in the prevention of sarcopenia. Am J Clin Nutr.

[33] Calvani R, Picca A, Coelho-Júnior HJ (2023). Diet for the prevention and management of sarcopenia. Metabolism.

[34] Huang H, Chen Z, Chen L (2022). Nutrition and sarcopenia: current knowledge domain and emerging trends. Front Med (Lausanne).

[35] Granic A, Cooper R, Robinson SM (2024). Myoprotective whole foods, muscle health and sarcopenia in older adults. Curr Opin Clin Nutr Metab Care.

[36] Chen LK, Arai H, Assantachai P (2022). Roles of nutrition in muscle health of community-dwelling older adults: evidence-based expert consensus from Asian Working Group for Sarcopenia. J Cachexia Sarcopenia Muscle.

[37] Ispoglou T, Witard OC, Duckworth LC (2021). The efficacy of essential amino acid supplementation for augmenting dietary protein intake in older adults: implications for skeletal muscle mass, strength and function. Proc Nutr Soc.

[38] Murphy CH, McCarthy SN, Roche HM (2023). Nutrition strategies to counteract sarcopenia: a focus on protein, LC n-3 PUFA and precision nutrition. Proc Nutr Soc.

[39] Campbell WW, Deutz NEP, Volpi E (2023). Nutritional interventions: dietary protein needs and influences on skeletal muscle of older adults. J Gerontol A Biol Sci Med Sci.

[40] Cuyul-Vásquez I, Pezo-Navarrete J, Vargas-Arriagada C (2023). Effectiveness of whey protein supplementation during resistance exercise training on skeletal muscle mass and strength in older people with sarcopenia: a systematic review and meta-analysis. Nutrients.

[41] Hengeveld LM, de Goede J, Afman LA (2022). Health effects of increasing protein intake above the current population reference intake in older adults: a systematic review of the Health Council of the Netherlands. Adv Nutr.

[42] Gielen E, Beckwée D, Delaere A (2021). Nutritional interventions to improve muscle mass, muscle strength, and physical performance in older people: an umbrella review of systematic reviews and meta-analyses. Nutr Rev.

[43] Shi Y, Tang Y, Stanmore E (2023). Non-pharmacological interventions for community-dwelling older adults with possible sarcopenia or sarcopenia: a scoping review. Arch Gerontol Geriatr.

[44] Ren Y, Lu A, Wang B (2023). Nutritional intervention improves muscle mass and physical performance in the elderly in the community: a systematic review and meta-analysis. Life (Basel).

[45] Coelho-Júnior HJ, Calvani R, Tosato M (2022). Protein intake and physical function in older adults: a systematic review and meta-analysis. Ageing Res Rev.

[46] Kamińska MS, Rachubińska K, Grochans S (2023). The impact of whey protein supplementation on sarcopenia progression among the elderly: a systematic review and meta-analysis. Nutrients.

[47] Ten Haaf DSM, Nuijten MAH, Maessen MFH (2018). Effects of protein supplementation on lean body mass, muscle strength, and physical performance in nonfrail community-dwelling older adults: a systematic review and meta-analysis. Am J Clin Nutr.

[48] Wirth J, Hillesheim E, Brennan L (2020). The role of protein intake and its timing on body composition and muscle function in healthy adults: a systematic review and meta-analysis of randomized controlled trials. J Nutr.

[49] Nasimi N, Sohrabi Z, Nunes EA (2023). Whey protein supplementation with or without vitamin D on sarcopenia-related measures: a systematic review and meta-analysis. Adv Nutr.

[50] Tagawa R, Watanabe D, Ito K (2020). Dose-response relationship between protein intake and muscle mass increase: a systematic review and meta-analysis of randomized controlled trials. Nutr Rev.

[51] Kirwan RP, Mazidi M, García C (2022). Protein interventions augment the effect of resistance exercise on appendicular lean mass and handgrip strength in older adults: a systematic review and meta-analysis of randomized controlled trials. Am J Clin Nutr.

[52] Li ML, Zhang F, Luo HY (2024). Improving sarcopenia in older adults: a systematic review and meta-analysis of randomized controlled trials of whey protein supplementation with or without resistance training. J Nutr Health Aging.

[53] Martin-Cantero A, Reijnierse EM, Gill BMT (2021). Factors influencing the efficacy of nutritional interventions on muscle mass in older adults: a systematic review and meta-analysis. Nutr Rev.

[54] Whaikid P, Piaseu N (2024). The effectiveness of protein supplementation combined with resistance exercise program among community-dwelling older adults with sarcopenia: s systematic review and meta-analysis. Epidemiol Health.

[55] Liao CD, Huang SW, Chen HC (2024). Comparative efficacy of different protein supplements on muscle mass, strength, and physical indices of sarcopenia among community-dwelling, hospitalized or institutionalized older adults undergoing resistance training: a network meta-analysis of randomized controlled trials. Nutrients.

[56] Thornton M, Sim M, Kennedy MA (2024). Nutrition interventions on muscle-related components of sarcopenia in females: a systematic review of randomized controlled trials. Calcif Tissue Int.

[57] Ganapathy A, Nieves JW (2020). Nutrition and sarcopenia-what do we know?. Nutrients.

[58] Nishimura Y, Højfeldt G, Breen L (2023). Dietary protein requirements and recommendations for healthy older adults: a critical narrative review of the scientific evidence. Nutr Res Rev.

[59] Institute of Medicine (2005). Dietary Reference Intakes for Energy, Carbohydrate, Fiber, Fat, Fatty Acids, Cholesterol, Protein, and Amino Acids.

[60] Westbury LD, Syddall HE, Fuggle NR (2020). Long-term rates of change in musculoskeletal aging and body composition: findings from the Health, Aging and Body Composition Study. Calcif Tissue Int.

[61] Soenen S, Rayner CK, Jones KL (2016). The ageing gastrointestinal tract. Curr Opin Clin Nutr Metab Care.

[62] Ho KC, Gupta P, Fenwick EK (2022). Association between age-related sensory impairment with sarcopenia and its related components in older adults: a systematic review. J Cachexia Sarcopenia Muscle.

[63] Porter J, Nguo K, Collins J (2019). Total energy expenditure measured using doubly labeled water compared with estimated energy requirements in older adults (≥ 65 years): analysis of primary data. Am J Clin Nutr.

[64] Cooper JA, Manini TM, Paton CM (2013). Longitudinal change in energy expenditure and effects on energy requirements of the elderly. Nutr J.

[65] Cox NJ, Morrison L, Ibrahim K (2020). New horizons in appetite and the anorexia of ageing. Age Ageing.

[66] Dodds RM, Granic A, Robinson SM (2020). Sarcopenia, long-term conditions, and multimorbidity: findings from UK Biobank participants. J Cachexia Sarcopenia Muscle.

[67] Ye L, Liang R, Liu X (2023). Frailty and sarcopenia: a bibliometric analysis of their association and potential targets for intervention. Ageing Res Rev.

[68] Aragon AA, Tipton KD, Schoenfeld BJ (2023). Age-related muscle anabolic resistance: inevitable or preventable?. Nutr Rev.

[69] Wall BT, Gorissen SH, Pennings B (2015). Aging is accompanied by a blunted muscle protein synthetic response to protein ingestion. PLoS One.

[70] Kramer IF, Verdijk LB, Hamer HM (2017). Both basal and post-prandial muscle protein synthesis rates, following the ingestion of a leucine-enriched whey protein supplement, are not impaired in sarcopenic older males. Clin Nutr.

[71] Bauer J, Biolo G, Cederholm T (2013). Evidence-based recommendations for optimal dietary protein intake in older people: a position paper from the PROT-AGE Study Group. J Am Med Dir Assoc.

[72] Hengeveld LM, Boer JMA, Gaudreau P (2020). Prevalence of protein intake below recommended in community-dwelling older adults: a meta-analysis across cohorts from the PROMISS consortium. J Cachexia Sarcopenia Muscle.

[73] Berner LA, Becker G, Wise M (2013). Characterization of dietary protein among older adults in the United States: amount, animal sources, and meal patterns. J Acad Nutr Diet.

[74] Bailey HM, Stein HH (2019). Can the digestible indispensable amino acid score methodology decrease protein malnutrition. Anim Front.

[75] Domić J, Grootswagers P, van Loon LJC (2022). Perspective: vegan diets for older adults? A perspective on the potential impact on muscle mass and strength. Adv Nutr.

[76] Lynch H, Johnston C, Wharton C (2018). Plant-based diets: considerations for environmental impact, protein quality, and exercise performance. Nutrients.

[77] Berrazaga I, Micard V, Gueugneau M (2019). The role of the anabolic properties of plant-versus animal-based protein sources in supporting muscle mass maintenance: a critical review. Nutrients.

[78] Gorissen SHM, Witard OC (2018). Characterising the muscle anabolic potential of dairy, meat and plant-based protein sources in older adults. Proc Nutr Soc.

[79] Duan Y, Li F, Li Y (2016). The role of leucine and its metabolites in protein and energy metabolism. Amino Acids.

[80] Schiaffino S, Reggiani C, Akimoto T (2021). Molecular mechanisms of skeletal muscle hypertrophy. J Neuromuscul Dis.

[81] Cholewa JM, Dardevet D, Lima-Soares F (2017). Dietary proteins and amino acids in the control of the muscle mass during immobilization and aging: role of the MPS response. Amino Acids.

[82] Rondanelli M, Nichetti M, Peroni G (2021). Where to find Leucine in food and how to feed elderly with sarcopenia in order to counteract loss of muscle mass: practical advice. Front Nutr.

[83] Granic A, Dismore L, Hurst C (2020). Myoprotective whole foods, muscle health and sarcopenia: a systematic review of observational and intervention studies in older adults. Nutrients.

[84] Burd NA, Beals JW, Martinez IG (2019). Food-first approach to enhance the regulation of post-exercise skeletal muscle protein synthesis and remodeling. Sports Med.

[85] Aoyama S, Nakahata Y, Shinohara K (2021). Chrono-nutrition has potential in preventing age-related muscle loss and dysfunction. Front Neurosci.

[86] Mao Z, Cawthon PM, Kritchevsky SB (2023). The association between chrononutrition behaviors and muscle health among older adults: the study of muscle, mobility and aging. Aging Cell.

[87] Hudson JL, Iii REB, Campbell WW (2020). Protein distribution and muscle-related outcomes: does the evidence support the concept?. Nutrients.

[88] Khaing IK, Tahara Y, Chimed-Ochir O (2024). Effect of breakfast protein intake on muscle mass and strength in adults: a scoping review. Nutr Rev.

[89] Hengeveld LM, Chevalier S, Visser M (2021). Prospective associations of protein intake parameters with muscle strength and physical performance in community-dwelling older men and women from the Quebec NuAge cohort. Am J Clin Nutr.

[90] Mendonça N, Hengeveld LM, Visser M (2021). Low protein intake, physical activity, and physical function in European and North American community-dwelling older adults: a pooled analysis of four longitudinal aging cohorts. Am J Clin Nutr.

[91] Wu PY, Huang KS, Chen KM (2021). Exercise, nutrition, and combined exercise and nutrition in older adults with sarcopenia: a systematic review and network meta-analysis. Maturitas.

[92] Tu DY, Kao FM, Tsai ST (2021). Sarcopenia among the elderly population: a systematic review and meta-analysis of randomized controlled trials. Healthcare (Basel).

[93] EFSA NDA Panel (EFSA Panel on Dietetic Products, Nutrition and Allergies) (2012). Scientific opinion on Dietary Reference Values for protein. EFSA J.

[94] Granic A, Hurst C, Dismore L (2020). Milk for skeletal muscle health and sarcopenia in older adults: a narrative review. Clin Interv Aging.

[95] Besora-Moreno M, Llauradó E, Valls RM (2022). Antioxidant-rich foods, antioxidant supplements, and sarcopenia in old-young adults ≥ 55 years old: a systematic review and meta-analysis of observational studies and randomized controlled trials. Clin Nutr.

[96] Park SJ, Park J, Won CW (2022). The inverse association of sarcopenia and protein-source food and vegetable intakes in the Korean Elderly: the Korean Frailty and Aging Cohort Study. Nutrients.

[97] Perälä MM, von Bonsdorff MB, Männistö S (2017). The healthy Nordic diet predicts muscle strength 10 years later in old women, but not old men. Age Ageing.

[98] Martel J, Ojcius DM, Ko YF (2019). Hormetic effects of phytochemicals on health and longevity. Trends Endocrinol Metab.

[99] Alì S, Davinelli S, Accardi G (2021). Healthy ageing and Mediterranean diet: a focus on hormetic phytochemicals. Mech Ageing Dev.

[100] Yousefzadeh MJ, Zhu Y, McGowan SJ (2018). Fisetin is a senotherapeutic that extends health and lifespan. EBioMedicine.

[101] Putra C, Konow N, Gage M (2021). Protein source and muscle health in older adults: a literature review. Nutrients.

[102] Bagherniya M, Mahdavi A, Shokri-Mashhadi N (2022). The beneficial therapeutic effects of plant-derived natural products for the treatment of sarcopenia. J Cachexia Sarcopenia Muscle.

[103] Fougere B, van Kan GA, Vellas B (2018). Redox systems, antioxidants and sarcopenia. Curr Protein Pept Sci.

[104] Prokopidis K, Mazidi M, Sankaranarayanan R (2023). Effects of whey and soy protein supplementation on inflammatory cytokines in older adults: a systematic review and meta-analysis. Br J Nutr.

[105] Wallace TC, Bailey RL, Blumberg JB (2020). Fruits, vegetables, and health: a comprehensive narrative, umbrella review of the science and recommendations for enhanced public policy to improve intake. Crit Rev Food Sci Nutr.

[106] Rosell M, Fadnes LT (2024). Vegetables, fruits, and berries – a scoping review for Nordic Nutrition Recommendations 2023. Food Nutr Res.

[107] Blumfield M, Mayr H, De Vlieger N (2022). Should we ‘eat a rainbow’? An umbrella review of the health effects of colorful bioactive pigments in fruits and vegetables. Molecules.

[108] Hu FB (2024). Diet strategies for promoting healthy aging and longevity: an epidemiological perspective. J Intern Med.

[109] Food and Agriculture Organization of United Nations (2016). Plates, Pyramids, Planet Developments in National Healthy and Sustainable Dietary Guidelines: A State of Play Assessment.

[110] Chung HY, Kim HJ, Kim KW (2002). Molecular inflammation hypothesis of aging based on the anti-aging mechanism of calorie restriction. Microsc Res Tech.

[111] Schmauck-Medina T, Molière A, Lautrup S (2022). New hallmarks of ageing: a 2022 Copenhagen ageing meeting summary. Aging (Albany NY).

[112] López-Otín C, Blasco MA, Partridge L (2023). Hallmarks of aging: an expanding universe. Cell.

[113] Baechle JJ, Chen N, Makhijani P (2023). Chronic inflammation and the hallmarks of aging. Mol Metab.

[114] Franceschi C, Campisi J (2014). Chronic inflammation (inflammaging) and its potential contribution to age-associated diseases. J Gerontol A Biol Sci Med Sci.

[115] Jimenez-Gutierrez GE, Martínez-Gómez LE, Martínez-Armenta C (2022). Molecular mechanisms of inflammation in sarcopenia: diagnosis and therapeutic update. Cells.

[116] Wang T (2022). Searching for the link between inflammaging and sarcopenia. Ageing Res Rev.

[117] Shivappa N, Steck SE, Hurley TG (2014). Designing and developing a literature-derived, population-based dietary inflammatory index. Public Health Nutr.

[118] Su Y, Yeung SSY, Chen YM (2022). The associations of dietary inflammatory potential with musculoskeletal health in Chinese community-dwelling older people: the Mr. OS and Ms. OS (Hong Kong) Cohort Study. J Bone Miner Res.

[119] Diao H, Yan F, He Q (2023). Association between Dietary Inflammatory Index and Sarcopenia: a meta-analysis. Nutrients.

[120] Xie H, Wang H, Wu Z (2023). The association of dietary inflammatory potential with skeletal muscle strength, mass, and sarcopenia: a meta-analysis. Front Nutr.

[121] García-Esquinas E, Rahi B, Peres K (2016). Consumption of fruit and vegetables and risk of frailty: a dose-response analysis of 3 prospective cohorts of community-dwelling older adults. Am J Clin Nutr.

[122] Sim M, Blekkenhorst LC, Lewis JR (2018). Vegetable and fruit intake and injurious falls risk in older women: a prospective cohort study. Br J Nutr.

[123] Ghoreishy SM, Asoudeh F, Jayedi A (2021). Fruit and vegetable intake and risk of frailty: a systematic review and dose response meta-analysis. Ageing Res Rev.

[124] Ribeiro SM, Morley JE, Malmstrom TK (2016). Fruit and vegetable intake and physical activity as predictors of disability risk factors in African-American middle-aged individuals. J Nutr Health Aging.

[125] Sabia S, Elbaz A, Rouveau N (2014). Cumulative associations between midlife health behaviors and physical functioning in early old age: a 17-year prospective cohort study. J Am Geriatr Soc.

[126] Perälä MM, von Bonsdorff M, Männistö S (2016). A healthy Nordic diet and physical performance in old age: findings from the longitudinal Helsinki Birth Cohort Study. Br J Nutr.

[127] Struijk EA, Guallar-Castillón P, Rodríguez-Artalejo F (2018). Mediterranean dietary patterns and impaired physical function in older adults. J Gerontol A Biol Sci Med Sci.

[128] Bruyère O, Reginster JY, Beaudart C (2022). Lifestyle approaches to prevent and retard sarcopenia: a narrative review. Maturitas.

[129] Ceglia L, Shea K, Rasmussen H (2023). A randomized study on the effect of dried fruit on acid-base balance, diet quality, and markers of musculoskeletal health in community dwelling adults. J Am Nutr Assoc.

[130] Craig JV, Bunn DK, Hayhoe RP (2017). Relationship between the Mediterranean dietary pattern and musculo-skeletal health in children, adolescents, and adults: systematic review and evidence map. Nutr Rev.

[131] McClure R, Villani A (2017). Mediterranean Diet attenuates risk of frailty and sarcopenia: new insights and future directions. J Cachexia Sarcopenia Muscle.

[132] Silva R, Pizato N, da Mata F (2018). Mediterranean diet and musculoskeletal-functional outcomes in community-dwelling older people: a systematic review and meta-analysis. J Nutr Health Aging.

[133] Bloom I, Shand C, Cooper C (2018). Diet quality and sarcopenia in older adults: a systematic review. Nutrients.

[134] Granic A, Sayer AA, Robinson SM (2019). Dietary patterns, skeletal muscle health, and sarcopenia in older adults. Nutrients.

[135] Jang EH, Han YJ, Jang SE (2021). Association between diet quality and sarcopenia in older adults: systematic review of prospective cohort studies. Life (Basel).

[136] Coelho-Júnior HJ, Trichopoulou A, Panza F (2021). Cross-sectional and longitudinal associations between adherence to Mediterranean diet with physical performance and cognitive function in older adults: a systematic review and meta-analysis. Ageing Res Rev.

[137] Papadopoulou SK, Detopoulou P, Voulgaridou G (2023). Mediterranean diet and sarcopenia features in apparently healthy adults over 65 years: a systematic review. Nutrients.

[138] Andreo-López MC, Contreras-Bolívar V, García-Fontana B (2023). The influence of the Mediterranean dietary pattern on osteoporosis and sarcopenia. Nutrients.

[139] Cailleaux PE, Déchelotte P, Coëffier M (2024). Novel dietary strategies to manage sarcopenia. Curr Opin Clin Nutr Metab Care.

[140] Dominguez LJ, Di Bella G, Veronese N (2021). Impact of Mediterranean diet on chronic non-communicable diseases and longevity. Nutrients.

[141] Mazza E, Ferro Y, Pujia R (2021). Mediterranean diet in healthy aging. J Nutr Health Aging.

[142] Trichopoulou A (2001). Mediterranean diet: the past and the present. Nutr Metab Cardiovasc Dis.

[143] Serra-Majem L, Tomaino L, Dernini S (2020). Updating the Mediterranean Diet Pyramid towards sustainability: focus on environmental concerns. Int J Environ Res Public Health.

[144] Hernández-Ruiz A, García-Villanova B, Guerra Hernández EJ (2015). Description of indexes based on the adherence to the Mediterranean dietary pattern: a review. Nutr Hosp.

[145] Davis C, Bryan J, Hodgson J (2015). Definition of the Mediterranean diet; a literature review. Nutrients.

[146] Gholami F, Bahrampour N, Samadi M (2023). The association of dietary acid load (DAL) with estimated skeletal muscle mass and bone mineral content: a cross-sectional study. BMC Nutr.

[147] Faure AM, Fischer K, Dawson-Hughes B (2027). Gender-specific association between dietary acid load and total lean body mass and its dependency on protein intake in seniors. Osteoporos Int.

[148] Cacciatore S, Calvani R, Marzetti E (2023). Low adherence to Mediterranean diet is associated with probable sarcopenia in community-dwelling older adults: results from the Longevity Check-Up (Lookup) 7+ Project. Nutrients.

[149] Coelho-Júnior HJ, Calvani R, Picca A (2023). Combined aerobic training and Mediterranean diet is not associated with a lower prevalence of sarcopenia in Italian older adults. Nutrients.

[150] Mazza E, Ferro Y, Maurotti S (2024). Association of dietary patterns with sarcopenia in adults aged 50 years and older. Eur J Nutr.

[151] Hayes EJ, Granic A, Hurst C (2021). Older adults’ knowledge and perceptions of whole foods as an exercise recovery strategy. Front Nutr.

[152] Mahony LO, Shea EO, O’Connor EM (2023). ‘Good, honest food’: older adults’ and healthcare professionals’ perspectives of dietary influences and food preferences in older age in Ireland. J Hum Nutr Diet.

